# Pancreatic Intraductal Papillary Mucinous Neoplasms: A Narrative Review

**DOI:** 10.15388/Amed.2023.30.1.6

**Published:** 2023-02-27

**Authors:** Daniel Paramythiotis, Eleni Karlafti, Georgia Fotiadou, Maria Charalampidou, Anestis Karakatsanis, Aristeidis Ioannidis, Antonios Michalopoulos

**Affiliations:** First Propaedeutic Surgery Department, University General Hospital of Thessaloniki AHEPA, Aristotle University of Thessaloniki, 54634 Thessaloniki, Greece; Emergency Department, University General Hospital of Thessaloniki AHEPA, Aristotle University of Thessaloniki, 54634 Thessaloniki, Greece;; First Propaedeutic Department of Internal Medicine, University General Hospital of Thessaloniki AHEPA, Aristotle University of Thessaloniki, 54634 Thessaloniki, Greece; First Propaedeutic Surgery Department, University General Hospital of Thessaloniki AHEPA, Aristotle University of Thessaloniki, 54634 Thessaloniki, Greece; First Propaedeutic Surgery Department, University General Hospital of Thessaloniki AHEPA, Aristotle University of Thessaloniki, 54634 Thessaloniki, Greece; First Propaedeutic Surgery Department, University General Hospital of Thessaloniki AHEPA, Aristotle University of Thessaloniki, 54634 Thessaloniki, Greece; First Propaedeutic Surgery Department, University General Hospital of Thessaloniki AHEPA, Aristotle University of Thessaloniki, 54634 Thessaloniki, Greece; First Propaedeutic Surgery Department, University General Hospital of Thessaloniki AHEPA, Aristotle University of Thessaloniki, 54634 Thessaloniki, Greece

**Keywords:** diagnostic imaging, guidelines, resection

## Abstract

**Introduction:**

Intraductal papillary mucinous neoplasms (IPMNs) are the most frequent cystic pancreatic neoplasm. They derive from the main pancreatic duct or branch ducts.

**Aim:**

This narrative review aims to present and compare the current guidelines on the management of IPMNs.

**Materials and methods:**

We reviewed the most important scientific literature on the management of IPMNs.

**Discussion:**

The clinical presentation of IPMNs is usually nonspecific; common symptoms are abdominal pain, weight loss, and jaundice. There are no sex differences, and the incidence increases with age. It is considered a premalignant lesion associated with synchronous or metachronous carcinomas. Multifocal sites within the pancreas and the presence of solid components, like mural nodules, are predictive factors for developing malignancy. Magnetic resonance imaging (MRI) is the imaging technique of choice. However, computed tomography (CT) and endoscopic ultrasound (EUS) with fine-needle aspiration (FNA) can also contribute to the diagnosis. Resection is the optimal treatment for IPMNs that arise from the main duct, while several indications are suggested for the surgery on IPMNs of branch ducts.

**Conclusion:**

The decision on surgery is not always a simple task and should be based on high-risk features of the neoplasm. In any case, re-examination and follow-up are highly recommended.

## Santrauka.

**Įvadas:**

Intraduktaliniai papiliniai mucininiai navikai (IPMN) yra dažniausios cistinės kasos neoplazmos, einančios nuo pagrindinio kasos latako ar nuo atšakinių latakų.

**Tikslas:**

Šios apžvalgos tikslas yra pristatyti ir palyginti šiuolaikines gaires, kaip spręsti su IPMN susijusias situacijas.

**Medžiaga ir metodai:**

Apžvelgėme svarbiausią mokslinę literatūrą, aptariančią IPMN situacijos sprendimus.

**Aptarimas:**

Klinikinė IPMN raiška įprastai nepasižymi jokiomis išskirtinėmis savybėmis; bendrieji simptomai yra pilvo skausmas, svorio praradimas ir gelta. Dėl lyties skirtumo nepastebima. Kuo vyresnė populiacijos grupė, tuo dažniau jai pasireiškia IPMN. Tai yra laikoma ikivėžiniu sveikatos sutrikimu, susijusiu su sinchroninėmis ar metachroninėmis karcinomomis. Piktybiškumo prognostiniai veiksniai yra daugybiniai židiniai kasoje ir kietųjų komponentų, tokių kaip sienelių mazgeliai, apraiškos. Tokioje situacijoje optimali technika yra magnetinio rezonanso (MRI) tyrimas. Tačiau diagnozei nustatyti gali padėti ir kompiuterinė tomografija (CT) bei endoskopinis ultragarsas (EUS) su aspiracija smulkia adata (FNA). Rezekcija yra optimalus iš pagrindinio latako išsivysčiusiems IPMN gydyti, tačiau iš šoninių latakų išsivysčiusių IPMN gydymo operacijų pasirinkimui rekomenduojama atsižvelgti į keletą indikacijų.

**Išvada:**

Apsispręsti dėl operacijos ne visada paprasta. Sprendimą reikėtų priimti, atsižvelgiant į didelio rizikos veiksnio neoplazmos savybes. Kad ir koks būtų priimtas sprendimas, labai rekomenduojama pakartotinė apžiūra ir tolesnis stebėjimas.

## Introduction

Intraductal papillary mucinous neoplasms (IPMNs) are the most common cystic pancreatic neoplasms. They are considered to be premalignant lesions, characterized by mucus-producing papillary hyperplasia of the epithelium of the pancreatic duct [1]. As a result, the main pancreatic duct or/and its branch-ducts are dilated over 5 mm, either diffusely or segmentally, without an apparent obstructing cause [2]. In contrast to mucinous cystic neoplasms (MCNs), IPMNs have no cellular ovarian-type stroma [3].

Pancreatic cancer with mucus production was first presented in 1982 by Ohashi et al., and in 1989 Morohoshi used the term “intraductal mucinous neoplasm” for the first time [4,5]. IPMNs account for 10–13% of all pancreatic cysts, 25–50% of all cystic pancreatic neoplasms, and 1% of pancreatic carcinomas [6].

IPMNs are characterized as premalignant lesions since a coexisting pancreatic carcinoma was found in one out of three cases during their surgical resection [7]. IPMNs constitute one of the three major precursor lesions of pancreatic infiltrating cancer. The other precursor lesions are pancreatic intraepithelial neoplasia (PanIN) and mucinous cystic neoplasm (MCN) [8].

These neoplasms are more common in patients >60–70 years old, and there are no significant differences in the incidence between males and females, while they are frequently seen in the head of the pancreas (50%). The tail of the pancreas or the uncinate process are more uncommon locations (7% and 4%, respectively) [9]. Interestingly, a high ratio of these neoplasms (about 39%) is multifocal over the pancreas [3,9]. In addition, the development of IPMNs has been related to genetic syndromes, such as familial adenomatous polyposis (FAP), BRCA2-associated hereditary breast cancer, Peutz–Jeghers syndrome, Von Hippel–Lindau (VHL) syndrome, familial pancreatic cancer (FPC), and various autoimmune diseases [2,10]. A medical history of diabetes mellitus (DM) or family history of pancreatic ductal adenocarcinoma (PDAC) has also been related to IPMNs development [11].

IPMNs are macroscopically visible tumors, necessarily > 1 cm in size. Depending on the involvement grade of the main pancreatic duct, they are classified as IPMNs deriving from the main pancreatic duct (main-duct type IPMNs, MD-IPMNs), IPMNs of the branch-ducts (branch duct type IPMNs, BD-IPMNs), and mixed type IPMNs (MT-IPMNs) [12].

Due to the increasing incidence [7] and the high malignant potential it is essential to study these neoplasms. Thus, early identification of patients with IPMNs can achieve better outcomes [13], the management however is not always a simple task. The existing guidelines suggest in some cases different approaches and as a result, the final decision is difficult to be made. The need for precision medicine is now more obvious than ever.

## Aim

This narrative review focuses on IPMNs and, additionally, aims to present and compare the different existing guidelines on the diagnosis and treatment of such neoplasms.

## Materials and methods

Bibliographical searches were performed in PubMed over the last ten years for the terms “intraductal papillary mucinous neoplasms”, “histopathology”, “diagnosis”, “surgery”, “prognosis”, and “guidelines”. Non-English publications were excluded, as well as articles for which full text was unretrievable.

## Discussion

### Epidemiology

A histological examination of every pancreatic cystic neoplasm, either surgically excised or detected through imaging techniques, is necessary for the establishment of the diagnosis. Thus, these neoplasms’ exact incidence and prevalence are still not clearly known [13]. The incidence rate was reported to be 2.04 cases per 100000 in 2008, increasing with age. While the prevalence in the general population was calculated as up to 26 cases per 100000, it reached up to 99 cases per 100000 in a population over 60 years old [14]. Although the incidence tends to increase over time, according to SEER research data, the mortality rate due to pancreatic cancer is stable [15]. Thus, the increase in incidence is attributed to more frequent scanning and better-quality radiological screening techniques [13]. In another study for pancreatic cysts, IPMN was diagnosed in about half of the excised neoplasms (25% MD- and 23% BD-IPMN) [16]. Finally, in a 33-year study at the Massachusetts General Hospital analyzing data from 851 resected cystic pancreatic tumors, 38% of them were classified as IPMNs [17].

### Clinical presentation

IPMNs do not usually present with typical symptoms, or are even incidentally discovered during imaging for nonspecific symptoms [18]. In contrast to BD-IPMNs, MD-IPMNs may present with abdominal pain due to the obstruction of the main pancreatic duct by the overproduction of mucus or with mild or moderate, recurrent, episodes of acute pancreatitis [6].

**Figure 1.**
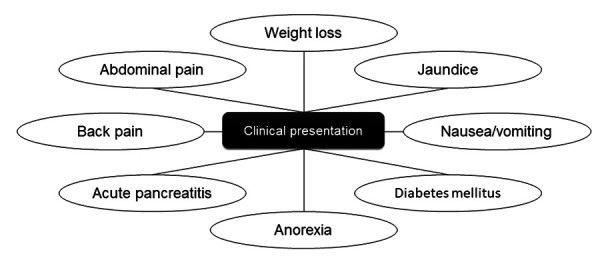
Clinical presentation of intraductal papillary mucinous neoplasms

Related symptomatology includes weight loss (20–40%), nausea or vomiting (11–21%), jaundice (15–20%), back pain (10%), or, more rarely, the onset of diabetes mellitus (DM) (Fig. 1). In addition, patients may develop persistent hyperamylasemia secondary to the chronic exocrine pancreatic insufficiency, or jaundice, due to obstruction of the common bile duct [2,6]. The formation of fistulas between these neoplasms and adjacent organs, like the stomach, the duodenum, the pleura, the small intestine, or the colon, is rare [19].

### Disease course

IPMNs require individualized surveillance, even after their excision, due to their malignant potential on one hand, and on the other hand, due to a general “instability” of the pancreatic parenchyma, which could lead to the development of carcinoma in other pancreatic sites, either synchronous or metachronous [20]. Even in elderly patients, despite their limited life span allowing for carcinomas to arise, IPMNs diagnosed at this age are usually more prone to developing infiltrating lesions. Moreover, multifocal IPMNs constitute a risk factor for developing a synchronous pancreatic adenocarcinoma [21].

There are a lot of predictive factors linked to a high risk of malignant evolution, that determine also the formulation of guidelines for the management of IPMNs [22]. Although established data have proven that the risk of malignancy is higher in cases of MD-IPMNs and MT-IPMNs (mixed type), the timeline of these changes has not yet been determined [2,23]. In a recent study of 1369 patients with BD-IPMN, who were placed under surveillance for at least 3 years, only 0.9% of them (13 patients) finally developed a high-grade dysplasia or an infiltrating cancer [24]. In another study, Pergolini et al. calculated the risk of malignancy development at about 8%, after a minimum of a 10-year period of surveillance, supporting the notion that all patients (initially operated on for noninvasive IPMNs) that are fit to undergo surgical resection must be systematically observed for as long as they are still considered as low-risk patients for pancreatic surgery [25].

### Coexisting pancreatic adenocarcinoma

Apart from the inherent malignant potential of IPMNs, they may coexist with a synchronous or metachronous pancreatic adenocarcinoma [26]. More specifically, 4–7% of excised IPMNs are associated with synchronous and 11% with metachronous pancreatic adenocarcinoma of the remaining pancreatic parenchyma [13]. The presence of a synchronous adenocarcinoma is also a risk factor for the development of a metachronous pancreatic adenocarcinoma [27].

### Macroscopic findings

IPMNs typically show up as cystic lesions with coexisting dilatation of the main pancreatic duct or its branch ducts, accompanied by atrophy of the surrounding pancreatic parenchyma. Due to the cystic nature of the lesion, the presence of a solid part increases the risk of malignancy existence [1,28].

Mural nodules form from papillary aggregations or a more complex neoplastic mass. These nodules are more probable to be highly dysplastic than the other areas of the neoplasms [29]. Imaging techniques can also detect the presence of mural nodules and provide valuable data for further treatment since their detection requires surgical treatment of the neoplasm. Regardless, mural nodules in the macroscopic examination of some IPMNs specimens are characterized by acellular mucus-lumps or reactive polypoid masses, with eroding walls of this cystic neoplasm instead of neoplastic tissue. Therefore, it has been suggested that surgical treatment should not be indicated solely on the presence of mural nodules [30,31].

Macroscopic examination of IPMNs may also confirm their multifocality in 20–40% of the cases, which also aids the differential diagnosis from a mucinous cystic neoplasm of the pancreas, where multifocality is uncommon [29].

### Microscopic findings

IPMNs are classified into four histopathological subtypes: the intestinal, which represents 18–36% of all IPMNs, the pancreatobiliary subtype is recognized in 7–18% of the specimens, the oncocytic variety in 1–8%, and finally, the gastric subtype is recognized in 46–63% of these neoplasms [9]. The gastric subtype is most commonly found in BD-IPMNs (98%), while the intestinal subtype is usually seen in MD-IPMNs (73%) [32].

The epithelium of the gastric subtype resembles the gastric mucosal and produces the MUC5AC protein. The epithelial tissue of the intestinal subtype resembles the villous adenoma of the colon and contains goblet cells, which express the MUC2 protein and the CDX2 transcription factor. Moreover, the pancreatobiliary subtype has a complex structure consisting of cuboidal epithelial cells with enlarged nuclei that express the MUC1 protein. IPMNs of the oncocytic subtype demonstrate arborizing papillary adhesions and solid clusters of eosinophil cells. Most of them express the MUC6 and HepPar1 proteins. It is worth noting that several authors consider the oncocytic subtype to be a distinct type of ductal neoplasia rather than a subtype of IPMNs, while some neoplasms appear with mixed subtypes in the same specimen [32,33].

**Figure 2.**
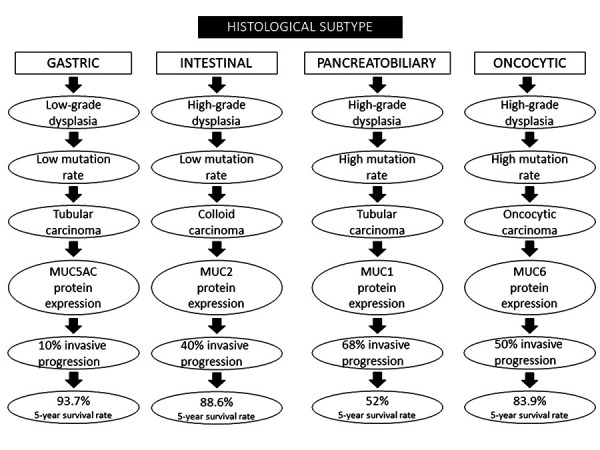
Differences between the subtypes of intraductal papillary mucinous neoplasms.

Several researchers have also identified a number of molecular genetic variations in IPMNs, some of which are in common with those seen in pancreatic adenocarcinomas, such as the mutations in KRAS, SMAD4, and TP53 genes [35]. The frequency of mutation in the KRAS gene is reported to be 40–87%. The most commonly mutated genes are KRAS, GNAS, and RNF43, while the GNAS codon 201 mutation is both the most frequent and specific mutation in IPMNs [32,35]. Overall, GNAS mutations are present more often in the intestinal subtype, whereas variations in the KRAS gene are usually found in the pancreatobiliary subtype (Fig. 2) [36].

The degree of dysplasia in the specimen is a crucial trait, which should always be reported, and depends on the atypia of the cells. In accordance with the WHO classification, low-grade dysplasia is defined based on the existence of a homogeneous layer of cylindrical cells with the nucleus located at the base of the cells and with no or minimal atypia. Atypia, polymorphism, and swelling of nuclei, with pseudostratification of cells, are present in intermediate-grade dysplasia, whereas marked atypia with complex structure and budding of abnormal epithelial cells in the lumen is characteristic of high-grade dysplasia [32,37]. Usually, different degrees of atypia appear in the same specimen; in this case, dysplasia is defined by the highest degree [34].

Invasive carcinomas that arise from IPMNs, present with heterogeneity in the cytomorphology, combining more than one type of epithelium, such as tubular (derived from the gastric subtype), colloid (intestinal subtype), and oncocytic [14].

Overall, the prognosis among the histological subtypes is better for the gastric one (93.7% 5-year survival rate) and worst for the pancreatobiliary one (52% 5-year survival rate). The exact percentage of invasive progression for every subtype is 10% for gastric, 40% for intestinal, 68% for pancreatobiliary, and 50% for oncocytic [38].

### Diagnostic imaging

IPMNs are diagnosed using high-resolution imaging methods as well as endoscopy. These methods aim to differentiate IPMNs from other pancreatic cystic neoplasms, identify their type, and determine risk factors for malignancy, while these methods are recommended to be utilized only for the period during which the patient remains at low surgical risk [39,40].

Every pancreatic cyst larger than 10 mm should be evaluated either with computed tomography (CT) or with gadolinium-enhanced magnetic resonance imaging (MRI) accompanied by magnetic resonance cholangiopancreatography (MRCP) [10,39]. MRI/MRCP is the imaging method of choice due to its greater sensitivity in detecting pancreatic neoplasms. Moreover, this method is optimal for identifying not only multiple nodules and whether there is communication between the cyst and the main pancreatic duct or not, but also risk factors for malignancy as well as the diameter of the main pancreatic duct [39,40]. According to recent research, diffusion-weighted MRI (DWI) could be beneficial for differential diagnosis between benign and malignant IPMNs, since diffusion restriction appears to be a radiological indicator for malignancy and invasiveness of IPMN [41].

Endoscopic ultrasound (EUS) is considered a second-line diagnostic tool after CT and MRI, as it is an invasive method. The operator’s experience determines the accuracy of EUS, but this technique offers the possibility of obtaining and analyzing cyst fluid through fine-needle aspiration (FNA). Endoscopic ultrasound can be beneficial in differentiating IPMNs from other pancreatic cysts such as serous cystadenoma, mucinous cystic neoplasm, and pseudocyst [42]. Levels of carcinoembryonic antigen (CEA), amylase/lipase, and glucose as measured in cyst fluid, as well as cytological findings of FNA, may increase the accuracy of diagnosis. CEA levels higher than 192 ng/ml seem to be able to distinguish mucinous from nonmucinous cysts, with a sensitivity of 38–78% and specificity of 63–99%. However, benign neoplasms cannot be distinguished from malignant ones based on CEA levels alone [20,39].

New technologies and methods such as next-generation sequencing (NGS) may improve significantly the differential diagnosis of pancreatic cysts. Identifying mutations in KRAS and GNAS genes may distinguish mucinous from nonmucinous cysts [43].

### Management of IPMNs

Surgical resection is, in general, advised for IPMNs, especially for main duct-IPMNs (MD-IPMNs) and mixed type-IPMNs (MT-IPMNs). Both of these neoplasms present a high risk of invasive cancer as well as a high disease-specific mortality rate if they are not appropriately treated [44]. Interestingly, malignancies that arise from branch-duct IPMNs (BD-IPMNs) are usually tubular carcinomas that resemble typical pancreatic adenocarcinoma and have a poor prognosis [45]. On the other hand, MD-IPMNs often mutate into colloid carcinoma with a better prognosis [46]. At present, timely surgery is the most efficacious treatment for both MD and MT-IPMNs [20]. To diminish the risk of complications, the guidelines recommend that these resections should be carried out by a skilled surgeon at a high-volume hospital for pancreatic operations. In addition, the American College of Gastroenterology (ACG) recommends that a joint decision by a council of doctors of various specialties (multidisciplinary team, MDT) should be required regarding the operation’s necessity [47]. In any case, advanced age and significant comorbidity are directly related to a high rate of complications and postoperative mortality, therefore, in such cases, palliative only measures should be also discussed [28].

On the contrary, the indications for surgery on BD-IPMNs differ in the various guidelines that have been published [20,28,31,47,48]. The International Association of Pancreatology (Fukuoka guidelines) reports as high-risk stigmata (HRS) the enhancing mural nodules greater than 5 mm, a diameter of the main pancreatic duct larger than 10 mm, and the presence of jaundice. Resection should be performed in patients with more than one of the above signs and at low surgical risk. Growth of the cyst more than 5 mm per year, a cyst diameter more than 3 cm with a thickened wall, a sudden change in the width of the pancreatic duct, an increase in serum levels of carbohydrate antigen 19-9 (CA 19-9), the presence of swollen lymph nodes, and the onset of pancreatitis are considered to be worrisome features (WF). In patients with WF, without HRS, endoscopic ultrasound should be performed to rule out a mural nodule as well as the main pancreatic duct’s involvement, and to obtain cytological material. On the occasion that endoscopic ultrasound cannot rule out malignancy, surgery should be performed [31].

The European guidelines define jaundice, a main pancreatic duct diameter of more than 10 mm, the presence of a solid or enhanced mural nodule, and a cytological examination positive for malignancy as absolute indications for resection. Relative indications for resection are an increase in cyst size of more than 5 mm per year, a cyst diameter of more than 4 cm, CA 19-9 levels of more than 37 U/mL, the onset of diabetes mellitus, and acute pancreatitis. In patients with low comorbidity, one relative indication may be enough for surgery, while in patients at high risk, at least two relative indications must be observed [20].

Since most IPMNs are located in the head of the pancreas, pancreaticoduodenectomy is the commonest procedure [49]. Biopsy from the edge of the remaining parenchyma is recommended so that the pancreatectomy may be expanded if neoplastic tissue is discovered [50]. In cases of increased risk of malignancy, such as in patients with a family history of pancreatic cancer or multiple lesions in the pancreatic parenchyma, total pancreatectomy (3–37% of patients) is recommended, with survival rates of 80% and 65% after the first and third year, respectively [7].

The above-mentioned surgical operations are associated with complications in 25% of all patients, with the most common being anastomotic leakage or stenosis, pancreatic fistula, intra-abdominal abscess, pancreatitis, pancreatic pseudocyst, cholangitis, delayed gastric emptying, ascites, diarrhoea, or nosocomial pneumonia. The in-hospital mortality rate is reported at 1.4%, while 30 days postoperatively, this rate arises to 2.7% [28].

In summary, revised Fukuoka and European guidelines recommend surgical resection for MD/MT-IPMNs, while the American College of Gastroenterology (ACG) suggests individualized management. On the other hand, there is no consensus on the indications for surgery on BD-IPMNs. According to Fukuoka, European, and ACG guidelines, either the presence of a solid mass or the dilatation of the main pancreatic duct is enough to decide on resection. On the contrary, the American Gastroenterological Association (AGA) proposes a more conservative management, with surgical management suggested when a main pancreatic duct dilatation coexists with a solid mass or concerning EUS/FNA findings [20,28,31,47,48]. Overall, many studies have shown that if dilatation of the main pancreatic duct is the only HRS detected in patients, surgery leads to overtreatment of the disease. However, AGA guidelines, that require the presence of one more risk factor for surgery to be advised, involve a risk of missing invasive cancer [38]. In that case, periodic, regular re-examination is recommended, provided that the patient remains at low surgical risk and consents to surgical resection [6]. Unfortunately, the frequency of follow-up investigations and the surveillance after resection are also controversial issues.

The comparisons between the guidelines are presented briefly in **Table 1** [28].

**Table 1.** Guidelines on management of intraductal papillary mucinous neoplasms (Copyright note. Modified table [20,28,31,47,48].).

	**Revised European guidelines (2018) [20]**	**Revised Fukuoka guidelines (2017) [31]**	**American College of Gastroenterology (2018) [47]**	**American Gastroenterological Association (2015) [48]**
**Diagnostic tools**	MRI/MRCP: method of choice CT: supplementary, to detect calcification, infiltration and metastases, recurrence of cancer after surgery EUS: complementary, in case of mural nodules FNA: in case of mural nodules, and septations, to differentiate mucinous from non-mucinous neoplasms CA 19-9 serum levels	MRI/MRCP: method of choice CT: supplementary EUS: in patients with worrisome features FNA: not recommended in patients with high-risk stigmata or worrisome features CA 19-9 serum levels	MRI/MRCP: method of choice CT: supplementary EUS/FNA: in case of unclear diagnose	MRI/MRCP: method of choice EUS/FNA: in case of high-risk features
**Indications for surgery on MD-/MT-IPMNs**	Patients at low surgical risk	Patients at low surgical risk with ≥ 1 high-risk stigmata	Multidisciplinary group for consideration of resection	Not mentioned
**High-risk factors and indications for surgery on BD-IPMNs**	Absolute indications: Solid tumor, Enhancing mural nodule ≥ 5 mm, Main pancreatic duct ≥ 10 mm, High-grade dysplasia/carcinoma in cytology, Jaundice Relative indications: Growth-rate ≥ 5 mm/year, Cyst diameter ≥ 4 cm, Enhancing mural nodule < 5 mm, Main pancreatic duct 5–9.9 mm, CA 19-9 serum levels ≥ 37 U/ml, New onset of diabetes mellitus, Acute pancreatitis Indications for surgery: ≥ 1 absolute indication or ≥ 1 relative indication in patients with low comorbidity or ≥ 2 relative indications in patients with high comorbidity	High-risk stigmata: Enhanced mural nodule > 5 mm, Main pancreatic duct > 10 mm, Obstructive jaundice Worrisome features: Growth-rate ≥ 5 mm/2 years, Cyst size ≥ 3 cm, Enhancing mural nodule < 5 mm, Enhanced thickened wall, Main pancreatic duct 5–9 mm, Change in main pancreatic duct caliber, Increased CA 19-9 serum levels, Acute Pancreatitis Indications for surgery: Cytology positive for high-grade dysplasia or ≥ 1 high-risk stigmata or ≥ 1 worrisome feature with mural nodule or main duct involvement	High-risk factors: High-grade dysplasia, Growth-rate > 3 mm/year, Cyst size ≥ 3 cm, Change in main pancreatic duct caliber, Main pancreatic duct > 5 mm, Mural nodule, Solid tumor, Increased CA 19-9 serum levels, Jaundice, Acute pancreatitis Indications for surgery: Multidisciplinary group for consideration of resection, in case of jaundice, solid mass, main duct involvement or main duct > 5 mm, high-grade dysplasia, mural nodule, cyst size ≥ 3 cm, or change in main pancreatic duct caliber	High-risk features: Cyst size > 3 cm, Solid tumor, Dilated main pancreatic duct Indications for surgery: Solid tumor, Main pancreatic duct ≥ 5 mm and/or concerning features on EUS/cytology positive for high-grade dysplasia
**Frequency of follow-up investigations**	CA 19-9, EUS and/or MRI every 6 months in the 1st year, then every year, as long as the patient remains at low surgical risk	< 1 cm: CT or MRI with MRCP within 6 months, then every 2 years, 1–2 cm: CT or MRI with MRCP every 6 months for 1 year, then every year for 2 years, then every 2 years, 2–3 cm: EUS or MRI with EUS in 3–6 months, then every 1 year, as long as the patient remains at low surgical risk	< 1 cm: MRI every 2 years, 1–2 cm: MRI every 1 year, 2–3 cm: MRI every 6–12 months, as long as the patient remains at low surgical risk	MRI with MRCP in 1 year, then every 2 years for 5 years
**Surveillance after resection**	Carcinoma: the same way as pancreatic cancer. High-grade dysplasia or MD-IPMNs: every 6 months for the first 2 years, and then every year. Low-grade dysplasia: the same way as non-resected.	Carcinoma: the same way as pancreatic cancer. In patients with a family history of pancreatic adenocarcinoma, high-grade dysplasia or non-intestinal subtype: every 6 months. In other patients: every 6–12 months.	Carcinoma: the same way as pancreatic cancer. High-grade dysplasia: every 6 months. Others: every 2 years.	Invasive cancer or dysplasia: every 2 years.

MRI/MRCP = Magnetic Resonance Imaging/Magnetic Resonance Cholangiopancreatography; CT = Computed Tomography; EUS = Endoscopic Ultrasound; FNA = Fine-Needle Aspiration; CA 19-9 = Carbohydrate antigen 19-9; MD-IPMNs = Main- Duct Intraductal Papillary Mucinous Neoplasms; MT-IPMNs = Mixed-Type Intraductal Papillary Mucinous Neoplasms; BD-IPMNs = Branch-Duct Intraductal Papillary Mucinous Neoplasms.

### The challenge of our era

Since there is no consensus on the management of IPMNs, surgical resection is not always a straightforward decision. The clinician with an interest in the management of IPMNs remains ill at ease, while choosing potentially between overtreatment and missing an invading carcinoma. Of course, individualized management, follow-up, and surveillance suggested by a multidisciplinary group of doctors at a hospital regarded as high-volume for pancreatic operations would be everyone’s suggestion. Nevertheless, there is a need for standard, worldwide accepted, evidence-based guidelines that provide a more accurate management plan concerning IPMNs. Diagnosing and treating these neoplasms adequately, while achieving a better quality of care and lower healthcare costs, is the field that future studies should focus on.

## Conclusion

Due to their high incidence and potential development of malignancy, it is of utmost importance to diagnose and treat pancreatic IPMNs early and properly. Their clinical presentation is usually nonspecific; therefore, high-resolution imaging methods, such as MRI/MRCP, CT, and EUS/FNA, should be performed according to guidelines to detect these neoplasms, as well as to provide follow-up. For most IPMNs (main-duct and mix-typed), resection is the treatment of choice. However, there are controversies between the guidelines for branch-duct IPMNs as issued by different associations. The high-risk features that indicate surgery, such as dilatation of the main pancreatic duct, vary among the different guidelines. As a result, it is sometimes difficult to decide on the management of each individual patient, especially considering the potential complications of such procedures, or the risk of these neoplasms to exhibit malignant behavior. Therefore, regular re-examination or follow-up should not be neglected.
